# Supplementary material for: Dynamic transcriptomic profiles of zebrafish gills in response to zinc supplementation
Source: BMC Genomics. 2010 Oct 11;11:553. doi: 10.1186/1471-2164-11-553 (PMC3091702; doi:10.1186/1471-2164-11-553)
Supplement: Additional file 2 — Interactive Direct Interaction Network representing the molecular interactions between zinc, copper, iron, calcium and proteins encoded by transcripts changed by zinc supplementation. Mini web-site containing index.html and hyperlinked pages in subdirectory describing a Direct Interaction Network automatically generated based on curated interactions contained within the proprietary PathwayArchitect database. Ovals represent proteins and the circles symbolize metal ions. Objects are coloured by their abundance in zebrafish at the time-point they were significantly different from the control is a scale from -4 fold (dark green) to +4 fold (dark red). Where significant differences were found at more than one time-point, the colour overlay shows expression at the first instance. Dark blue squares denote 'binding', and light blue squares 'expression'; green squares stand for 'regulation', green diamonds for 'metabolism', and green circles for 'promoter binding'. Arrow heads indicate directionality of the interaction where annotated. All nodes and edges can be further interrogated by selecting the relative area of the image. [file 1471-2164-11-553-S2.zip › PathwayArchitect Zn xs DIN/639432.html]

# REGULATION:

|  |  |
| --- | --- |
| Type | REGULATION |
| Effect | Positive |


---

|  |  |
| --- | --- |
| Score | 0 |


---

|  |  |
| --- | --- |
| Reference Count | 9 |


---

|  |  |
| --- | --- |
| Mechanism | Unknown |


---

|  |  |
| --- | --- |
| Reference:0 || Sentence | "Despite its higher net charge and reduced opportunities for favorable tertiary interactions, Ca(2+)-free rat beta-parvalbumin is more stable than rat alpha-parvalbumin." |
| PMID | 12974622 |
| Year | 2003 |
| Species | Rat |
| Journal | Biochemistry |
| RefScore | 1 |
| Source | PArchNLP |
  |
|  |  |


---

|  |  |
| --- | --- |
 Reference:1 || Sentence | "Analysis of the effects of calbindin D28k (370 microM) and parvalbumin (1 mM) on Ca2+ currents in the whole-cell voltage clamp mode, revealed that neither protein changed the rate of inactivation of the Ca2+ current or its rate of run-down. 5." |
| PMID | 8145149 |
| Year | 1993 |
| Species | Rat |
| Journal | J Physiol |
| RefScore | 2 |
| Source | PArchNLP |
  ||  |  |


---

|  |  |
| --- | --- |
 Reference:2 || Sentence | "Parvalbumin enhanced Ca2+ uptake activity of the light sarcoplasmic reticulum." |
| PMID | 8123046 |
| Year | 1994 |
| Journal | Biochem Biophys Res Commun |
| RefScore | 0 |
| Source | PArchNLP |
  ||  |  |


---

|  |  |
| --- | --- |
 Reference:3 || Sentence | "The interactions of pike parvalbumins are modulated by Ca2+ and Mg2+ binding to the protein and induce some changes in the physical properties of both the proteins and liposomes." |
| PMID | 3396853 |
| Year | 1988 |
| Species | Human |
| Journal | Gen Physiol Biophys |
| RefScore | 1 |
| Source | PArchNLP |
  ||  |  |


---

|  |  |
| --- | --- |
 Reference:4 || Sentence | "The most parsimonious trees show many parallel or back mutations in the evolution of many parvalbumin residues, although the residues responsible for Ca2+ binding are very well conserved." |
| PMID | 6599978 |
| Year | 1984 |
| Journal | Mol Biol Evol |
| RefScore | 0 |
| Source | PArchNLP |
  ||  |  |


---

|  |  |
| --- | --- |
 Reference:5 || Sentence | "Calbindin-D(28k) (CB), calretinin (CRT), and parvalbumin (PV) are high-affinity cytosolic calcium (Ca(2+)) binding proteins (CBP) that have been found to regulate intracellular calcium concentrations in neurons through their buffering capacity and to protect neurons from insults that induce elevations of intracellular Ca(2+)." |
| PMID | 12821392 |
| Year | 2003 |
| Species | Human |
| Journal | Exp Neurol |
| RefScore | 1 |
| Source | PArchNLP |
  ||  |  |


---

|  |  |
| --- | --- |
 Reference:6 || Sentence | "However, when EGTA-free solutions were used, calbindin D28k and parvalbumin significantly increased ATP-dependent Ca2+ uptake in erythrocyte as well as in enterocyte-derived membrane vesicles." |
| PMID | 7602380 |
| Year | 1995 |
| Species | Rat |
| Journal | J Nutr |
| RefScore | 0 |
| Source | PArchNLP |
  ||  |  |


---

|  |  |
| --- | --- |
 Reference:7 || Sentence | "In addition, ATP-dependent Ca2+ uptake in the presence of 0.2 microM calmodulin was further increased by parvalbumin in the absence but not in the presence of EGTA." |
| PMID | 7602380 |
| Year | 1995 |
| Species | Rat |
| Journal | J Nutr |
| RefScore | 0 |
| Source | PArchNLP |
  ||  |  |


---

|  |  |
| --- | --- |
 Reference:8 || Sentence | "Like PA, EDTA's contribution to muscle relaxation and Ca2+ sequestration was more clearly observed when the SR Ca-ATPase was inhibited." |
| PMID | 10049331 |
| Year | 1999 |
| Species | Rat |
| Journal | Biophys J |
| RefScore | 1 |
| Source | PArchNLP |
  |


---

|  |  |
| --- | --- |
